# The use of natural language processing in detecting and predicting falls within the healthcare setting: a systematic review

**DOI:** 10.1093/intqhc/mzad077

**Published:** 2023-09-27

**Authors:** Vincent Quoc-Nam Trinh, Steven Zhang, Joshua Kovoor, Aashray Gupta, Weng Onn Chan, Toby Gilbert, Stephen Bacchi

**Affiliations:** University of Adelaide, Adelaide, South Australia 5005, Australia; University of Adelaide, Adelaide, South Australia 5005, Australia; University of Adelaide, Adelaide, South Australia 5005, Australia; Queen Elizabeth Hospital, Adelaide, South Australia 5011, Australia; University of Adelaide, Adelaide, South Australia 5005, Australia; Gold Coast University Hospital, Gold Coast, Queensland 4215, Australia; Queen Elizabeth Hospital, Adelaide, South Australia 5011, Australia; Discipline of Ophthalmology and Visual Sciences, The University of Adelaide, Adelaide, South Australia 5005, Australia; Royal Adelaide Hospital, Adelaide, South Australia 5000, Australia; University of Adelaide, Adelaide, South Australia 5005, Australia; Northern Adelaide Local Health Network, Adelaide, South Australia 5112, Australia; Royal Adelaide Hospital, Adelaide, South Australia 5000, Australia; Flinders University, Adelaide, South Australia 5042, Australia

**Keywords:** predictive analytics, artificial intelligence, geriatrics

## Abstract

Falls are a common problem associated with significant morbidity, mortality, and economic costs. Current fall prevention policies in local healthcare settings are often guided by information provided by fall risk assessment tools, incident reporting, and coding data. This review was conducted with the aim of identifying studies which utilized natural language processing (NLP) for the automated detection and prediction of falls in the healthcare setting. The databases Ovid Medline, Ovid Embase, Ovid Emcare, PubMed, CINAHL, IEEE Xplore, and Ei Compendex were searched from 2012 until April 2023. Retrospective derivation, validation, and implementation studies wherein patients experienced falls within a healthcare setting were identified for inclusion. The initial search yielded 2611 publications for title and abstract screening. Full-text screening was conducted on 105 publications, resulting in 26 unique studies that underwent qualitative analyses. Studies applied NLP towards falls risk factor identification, known falls detection, future falls prediction, and falls severity stratification with reasonable success. The NLP pipeline was reviewed in detail between studies and models utilizing rule-based, machine learning (ML), deep learning (DL), and hybrid approaches were examined. With a growing literature surrounding falls prediction in both inpatient and outpatient environments, the absence of studies examining the impact of these models on patient and system outcomes highlights the need for further implementation studies. Through an exploration of the application of NLP techniques, it may be possible to develop models with higher performance in automated falls prediction and detection.

## Introduction

Falls in hospitalized patients represent a leading cause of mortality and morbidity, with falls accounting for the leading cause of injury-related deaths in patients aged over 70 [1]. In the cohort, falls were second only to road injuries in death due to unintentional injuries [2].

The accurate prediction and early detection of falls reduces the incidence and severity of complications [3]. Current falls prevention policies are implemented based upon risk assessment tools, incident reporting, clinical notes, and coding. However, analysis of medical records is time intensive, utilizing data of variable quality and subjective judgement. As the factors contributing to falls are multifactorial [4], this difficulty in recording quality data has contributed to further difficulties in detecting and predicting the occurrence of falls based on retrospective clinical data.

Natural language processing (NLP) offers the ability to analyse unstructured clinical text in the form of patient electronic medical records, and has been used in conjunction with statistical or Machine Learning (ML) models to create classifiers to detect or predict the presence of an outcome of interest [5]. For this to occur, text data must be provided in a training sample with labelled cases for the unknown outcome. This labelling and analysis can occur at a sentence, paragraph, or document level. These documents then undergo text preprocessing to remove unnecessary characters and stop words, following which the preprocessed text is converted into numerical representations for analysis. These features may be used in combination with structured numerical or categorical input variables. The dataset is then split into training and validation datasets with the model being validated and optimized on a number of performance metrics.

The application of NLP towards falls detection and prediction has evolved considerably over time. It was seen that earlier models consisted of statistical text mining and simple rule-based algorithms prior to the introduction of classical ML methods such as linear or logistic regression, support vector machines (SVM), and random forest (RF). Models now take more complex architectures with Deep Learning (DL) approaches that utilize convoluted neural network (CNN) and recurrent neural network (RNN) variants becoming more common. Recently, the widespread use of transformers and self-attention mechanisms offers many advantages over traditional sequence-to-sequence approaches [6].

Currently, there is large variability in the current NLP literature with regard to fall prediction or detection in terms of: sources of unstructured text data, data preprocessing techniques, model architecture, classifier training, and assessment of model performance. To date, there have been no systematic reviews examining the applications of NLP in detecting and predicting falls in healthcare settings.

This systematic review aimed to synthesize the current literature with regard to the use of NLP in falls detection and prevention for a healthcare setting.

The primary research questions include:

1. In what ways have NLP algorithms been applied to falls detection and prediction?
2. What sources of unstructured text data are utilized as data information and what were their characteristics?
3. What data annotation methodology and preprocessing techniques are used to convert unstructured text data to training data for ML algorithms?
4. How was the ML algorithm trained and developed?
5. What architectures were utilized for ML-NLP algorithms?
6. How effective were ML-NLP algorithms at identifying and predicting clinical outcomes?
7. Have implementation studies been conducted and what were the findings?

### PICO summary

Population: Falls within a healthcare setting, Intervention: NLP-ML, Comparison: Fall risk assessment tools, manual chart review, Outcomes: Detection and prediction of falls.

## Methods

This systematic review was conducted in accordance with the Preferred Reporting Items for Systematic Reviews and Meta-Analyses (PRISMA) guidelines [7]. The review protocol was prospectively registered with the International Prospective Register of Systematic Reviews (PROSPERO) (Registration ID: CRD42022323742).

### Search strategy

Searches of Ovid Medline, Ovid Embase, Ovid Emcare, PubMed, CINAHL, IEEE Xplore, and Ei Compendex databases were conducted from inception until April 2023. Relevant terms were identified through the MeSH/Emtree database. The full search strategy is outlined in Supplementary Information 1. Search results were exported to Covidence [8] where duplicate removal was performed. Two independent reviewers (V.T, S.Z) screened articles for inclusion based on title, abstract, and full text. All disagreements were resolved by consensus and through discussion with a third reviewer.

### Eligibility criteria

Studies utilizing NLP in conjunction with statistical and ML methods to detect or predict falls within a healthcare setting were included. Exclusion criteria included: (i) Non-English language, (ii) Wrong publication type (conference proceedings, abstracts), (iii) Published prior to 2012, (iv) Non-healthcare setting, (v) Falls not examined, and (vi) NLP not utilized or insufficient information on NLP methods.

### Data extraction and quality assessment

Data extraction was performed by two reviewers (V.T and S.Z) in duplicate using a priori data extraction template. This data extraction template was informed by the PRISMA guidelines, Cochrane Handbook for Systematic Review of Interventions [9], and the Transparent Reporting on a Multivariable Prediction Model for Individual Prognosis or Diagnosis (TRIPOD) checklist [10]. Discrepancies were resolved by discussion and adjudicated by a third reviewer.

The quality of included articles was assessed using the Guidelines for Developing and Reporting Machine Learning Predictive Models in Biomedical Research [11]. The modified quality assessment and data extraction templates can be found in Supplementary Information 2.

### Data synthesis

Qualitative and quantitative data were synthesized in narrative and tabular formats. A quantitative approach was not undertaken as it was anticipated that a high degree of heterogeneity would preclude meta-analyses. Comparative analyses were performed using R Statistical Software v4.2.2 [12].

## Results

**Figure 1:**
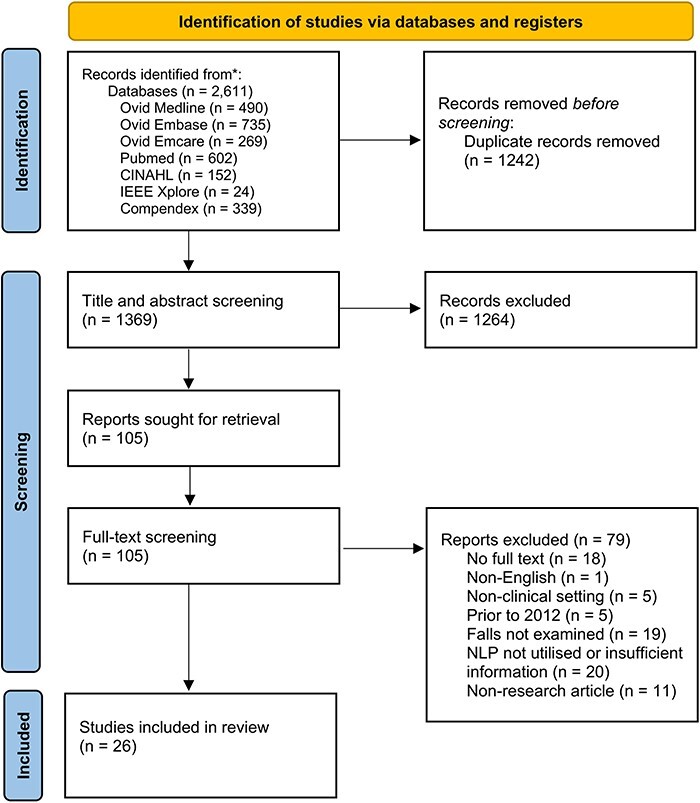
PRISMA flow diagram of included publications.

The search identified 2611 publications for retrieval with 1369 articles for title and abstract screening (Fig. 1). There was strong agreement between both reviewers (0.92 proportionate agreement, Cohen’s κ 0.51). A total of 105 publications underwent full-text screening with 26 meeting inclusion criteria [13–38]. Overall inter-rater reliability was high (proportionate agreement 0.87, Cohen’s κ 0.71).

### General characteristics

Articles originated from a variety of countries with publications from the United States, Japan, and Australia being the most numerous. Studies were all retrospective with the majority taking place within multi-centre inpatient hospital settings (Table 1). Outpatient settings included clinics, homecare-visits, primary care, and residential aged care facilities (RACF). One study examined the application of NLP towards falls within an ambulatory care setting [32].

**Table 1.** General characteristics of included publications.

Reference	Year	Country	NLP task	Study type	Setting	Number of centres	Participants
Anzaldi et al.	2017	United States	Detection	Derivation Validation	Inpatient (Emergency Department) Outpatient (Home, Clinic, RACF)	Multicentre	Age ≥65 years with health insurance coverage
Bates et al.	2016	United States	Detection	Derivation Validation	Outpatient (Clinic)	Multicentre	HIV-positive veterans with HIV-negative veteran matched controls
Bjarnadottir et al.	2018	United States	Risk Factor Identification	Derivation	Inpatient (Intensive Care Unit)	Single centre	Criteria not specified
Chen et al.	2015	United States	Detection	Derivation Validation	Not specified	Multicentre	Age ≥65 years with health insurance coverage
Dormosh et al.	2023	Netherlands	Risk Factor Identification Prediction	Derivation Validation	Outpatient (Primary Care)	Multicentre	Age ≥65 years
Fong et al.	2015	United States	Risk Factor Identification	Derivation	Inpatient (Hospital)	Multicentre	Criteria not specified
Fu et al.	2022	United States	Detection	Derivation Validation	Inpatient (Hospital)	Multicentre	Age ≥65 years
Kawazoe et al.	2022	Japan	Prediction	Derivation Validation Impact Analyses	Inpatient (Hospital)	Single centre	Age ≥65 years
Kharrazi et al.	2018	United States	Detection	Validation Impact Analyses	Inpatient (Emergency Department) Outpatient (Home, Clinic, RACF)	Multicentre	Age ≥65 years with health insurance coverage
Leurs at al.	2022	Netherlands	Risk Factor Identification	Derivation	Inpatient (Hospital)	Single centre	Age ≥70 years with case matched controls
Liu et al.	2021	Hong Kong	Severity Stratification	Derivation Validation	Inpatient (Hospital)	Multicentre	Hospitalized inpatients
Luther et al.	2015	United States	Detection	Derivation Validation	Inpatient (Hospital, Emergency Department) Outpatient (Clinic)	Multicentre	Patients receiving treatment for injuries or fall-related electronic codes with age matched controls
Martin et al.	2022	United States	Detection Prediction	Derivation Validation	Inpatient (Hospital) Outpatient (Clinic, Primary Care)	Multicentre	Diagnosis of chronic lung disease
McCart et al.	2013	United States	Detection	Derivation Validation (both internal and external)	Outpatient (Clinic)	Multicentre	Patients receiving treatment for injuries or fall-related electronic codes with age matched controls
Nakatani et al.	2020	Japan	Risk Factor Identification Detection Prediction	Derivation Validation	Inpatient (Hospital, Emergency Department)	Single centre	Hospitalized inpatients
Navathe et al.	2016	United States	Detection	Impact Analyses	Inpatient (Hospital)	Multicentre	Diagnosis of cardiovascular disease
Patterson et al.	2019	United States	Detection	Derivation Validation	Inpatient (Emergency Department)	Single centre	Age ≥65 years
Shiner et al.	2020	United States	Detection	Derivation Validation	Inpatient (Hospital)	Single centre	Veteran inpatients
Takase et al.	2023	Japan	Risk Factor Identification	Derivation	Inpatient (Hospital)	Multicentre	Hospitalized inpatients
Tohira et al.	2022	Australia	Detection	Derivation Validation	Ambulatory Care	Multicentre	Ambulatory care patients
Topaz et al.	2019	United Stated	Risk Factor Identification Detection Prediction	Derivation Validation	Outpatient (Home)	Single centre	Criteria not specified
Topaz et al.	2019	United Stated	Risk Factor Identification	Derivation	Outpatient (Home)	Single centre	Criteria not specified
Toyabe	2012	Japan	Detection	Derivation Validation	Inpatient (Hospital)	Single centre	Hospitalized inpatients
Toyabe	2015	Japan	Detection	Validation Impact Analyses	Inpatient (Hospital)	Single centre	Hospitalized inpatients
Wang et al.	2017	Australia	Detection	Derivation Validation (both internal and external)	Inpatient (Hospital)	Multicentre	Hospitalized inpatients
Wang et al.	2019	Australia	Detection	Derivation Validation (both internal and external)	Inpatient (Hospital)	Multicentre	Hospitalized inpatients

Patient demographics varied significantly with eight studies focusing on older patients (aged ≥65). Other subpopulations included veteran inpatients (two studies) and specific medical diagnoses: HIV (one study), chronic lung disease (one study), cardiovascular disease (one study), fall-related injury (two studies). Four studies examined case-matched controls.

Four overlapping categories were found in terms of NLP applications: (i) Falls risk factor identification (8 studies); (ii) Fall detection: identifying known falls occurring from clinical documents (18 studies); (iii) Fall prediction: identifying future falls from clinical documentation preceding the fall event (4 studies); and (iv) Fall severity stratification (1 study). No implementation studies were identified.

### Quality of included studies

Quality assessment of included publications yielded a mean score of 73.7% (range: 59.5%–88.2%) representing moderate adherence to checklist items (Supplementary Information 3). It was seen that publication quality was generally higher in more recent studies.

### Data sources and annotation

Both structured and unstructured data sources were utilized as sources of data information; seven studies utilized data from established clinical databases (Table 2). Medical progress notes formed the backbone of most unstructured data input (58% of studies), followed equally by incident reports and nursing notes (35% each). Data from previous admissions were incorporated into the NLP models of seven studies.

**Table 2.** Data sources and annotation methodology for included publications.

Reference	Structured data	Unstructured data	Historical patient data	Dataset source	Training data size^a^	Testing data size	Fall capture rate (%)	Number of annotators	Annotator knowledge	Inter-annotator agreement
Anzaldi et al.	EHR fields	Medical progress notes, nursing notes, discharge summaries, clinic notes, home-care notes, emails, transcribed phone calls	Yes	EHR data (Atrius Health)	24 420	13 200	Not specified	≥2	Clinician	Not specified
Bates et al.	NA	Imaging reports	Yes	Veterans Ageing Cohort Study Virtual Cohort (VACS-VC) database	5349	2939	Train:7.4 Test: 4.9	≥2	Domain Expert	0.93 (κ)
Bjarnadottir et al.	NA	Nursing Notes	No	Medical Information Mart for Intensive Care (MIMIC-III) dataset	1 046 053	NA	NA	NA	NA	NA
Chen et al.	ICD-9 codes	Medical progress notes	No	EHR data (Unspecified Centre)	74 673 sentences	45 148 sentences	Train: 0.5 Test: 0.42	Single	Clinician	NA
Dormosh et al.	Demographics, EHR fields, medications, medical diagnoses	Medical progress notes, allied health notes	No	Academic General Practitioners Network (AHA AMC) database	Not specified (Concatenated record)	Not specified (Concatenated record)	Not specified	Not specified	Not specified	Not specified
Fong et al.	EHR fields	Incident reports	No	Incident reporting system (multi- hospital)	49 859	NA	Train: 12	≥2	Domain Expert	0.79 (proportionate agreement)
Fu et al.	NA	EHR Freetext (not specified)	No	Mayo Clinic Biobank database	4357	1090	Train: 19.8 Test: 24.1	≥2	Trained Annotator	0.72 (concept level F1 score)
Kawazoe et al.	Demographics, laboratory values, ICD-10 codes, medical diagnoses	Incident reports, medical progress notes, nursing notes	Yes	EHR data (University of Tokyo Hospital)	61 560	10 386	Train: 2.4 Test: 2.2	Not specified	Not specified	Not specified
Kharrazi et al.	EHR fields, insurance claims data	Medical progress notes, nursing notes, discharge summaries, emails, transcribed phone calls	Yes	EHR data (Atrius Health)	24 420	13 200	Not specified	≥2	Clinician	Not specified
Leurs at al.	NA	Nursing notes	No	EHR data (Catharina Hospital, Netherlands)	362	NA	Train: 50.3 (case matched controls)	Not specified	Nursing staff	Not specified
Liu et al.	Structured incident report fields	Incident reports	No	Incident reporting system (multi-hospital)	978	244	100 (falls only)	≥2	Domain Expert	0.039 (divergence rate)
Luther et al.	NA	Incident reports, nursing notes, medical progress notes	No	EHR data (Veteran Health Administration)	6496	7.356	Not specified	≥2	Clinician	0.90 (κ)
Martin et al.	Demographics, laboratory values, vital signs	Medical progress notes	Yes	EHR data (University of Pennsylvania Health System)	724 sentences	586 sentences	Train: 1 Test: 0.7	≥2	Trained Annotator	Not specified
McCart et al.	NA	Medical progress notes	No	EHR data (Veteran Health Administration)	6641	19 369	Train: 18.2 Test: 19.7	≥2	Clinician	0.90 (κ)
Nakatani et al.	EHR fields	Nursing notes	No	EHR data (NTT Medical Centre, Tokyo)	Detection: 12 619 Prediction: 9094	Detection: 12 526 Prediction: 9813	Train: 72 Test: 78	Not specified	Not specified	Not specified
Navathe et al.	Demographics, laboratory values, ICD-9 codes, EHR fields, medications	Medical progress notes, discharge summaries, administrative claims	Yes	EHR data (multi-hospital)	500	600	Train: 15.6 Test: Not specified	≥2	Domain Expert	Not specified
Patterson et al.	NA	Medical progress notes	No	EHR data (Emergency department)	1084	500	Train: Not specified Test: 24	≥2	Trained Annotator	0.96 (κ)
Shiner et al.	NA	Medical progress notes	No	EHR data (Veterans Affairs Hospitals)	2730	1574	Train: 1.5 Test: 12.4	≥2	Domain Expert	0.88 (κ)
Takase et al.	Demographics, structured incident report fields, utilization statistics	Incident reports	No	Japanese Council for Quality Health Care (JCQHC) database	4176	NA	100 (falls only)	Not specified	Not specified	Not specified
Tohira et al.	Demographics, EHR fields, medical diagnoses, vital signs, treatment, dispatch priority, transport urgency	Paramedic care notes	No	Electronic patient care records (ePCR) database	6612	2835	Train: 12.9 Test: 12.8	Single	Clinician	NA
Topaz et al.	NA	Medical progress notes, nursing notes, allied health notes	No	EHR data (Homecare agency)	1704	750	Train: 4.4 Test: 9.5	≥2	Trained Annotator	0.84 (κ)
Topaz et al.	NA	Medical progress notes, nursing notes, allied health notes	No	EHR data (Homecare agency)	1 149 586	NA	Not specified	Not specified	Not specified	Not specified
Toyabe	Structured incident report fields	Incident reports, medical progress notes, discharge summaries, image order entries	Yes	HER Data & Incident Reporting system (Niigata University Hospital)	2590	234 014	Train: 10.7 Test: 0.12	Single	Clinician	NA
Toyabe	Demographics	Incident reports, medical progress notes	No	Incident reporting system (Niigata University Hospital)	NA	640 434	Test: 0.1	Not specified	Not specified	Not specified
Wang et al.	Structured incident report fields	Incident reports	No	Advanced Incident Management System (AIMS) Riskman	2860	6000	Train: 9.1 Test: 14.5	≥2	Domain Expert	0.93 (κ)
Wang et al.	Structured incident report fields	Incident reports	No	Advanced Incident Management System (AIMS) Riskman	2860	6000	Train: 9.1 Test: 14.5	≥2	Domain Expert	0.93 (κ)

a Data size is represented as document number unless stated otherwise.

Data sample size for the training set was compared on a document and sentence level, demonstrating significant variability. It was observed that on a document level for model training: two studies (7.7%) utilized 200–500 documents, one study (3.8%) utilized 501–1000 documents, two studies (7.7%) utilized 1001–2000 documents, six studies (23.1%) utilized 2001–5000 documents, four studies (15.4%) utilized 5001–10 000 documents, and seven studies (26.9%) utilized over 10 000 documents. When stratified by architecture, hybrid and text mining models were associated with the smallest (3031 ± 1876) and largest (350 197 ± 602 632) number of documents by training sample, respectively. Four studies did not report their data size in terms of document number (Supplementary Information 4).

The mean fall capture rate as defined by the proportion of documents labelled as a fall or containing falls-related information was 15.4% (range 0.5–72) and 14.5% (0.1–78) when compared across training and test sets, respectively. This was not reported in nine studies, and two studies examined falls patients only.

Data annotation methodology was reported in sufficient detail by 18 studies, with 15 studies (57.7%) utilizing two or more annotators and 3 studies (11.5%) utilizing a single annotator. It was found that annotators were diverse in clinical background with clinicians and domain experts being the most common (26.9% of studies each), followed by trained annotators (15.4%). The involvement of nursing staff in the annotation process was infrequent and occurred in one study. Inter-annotator agreement was reported by most studies with multiple annotators (11 of 15, 73.3%) with Cohen’s kappa most commonly utilized alongside proportionate agreement and divergence rates. Classification results were most often compared to a manually annotated corpus (MAC) with only two studies relying on initial reporter classification (IRC).

Common steps for data-cleaning involved case-conversion and the removal of punctuation and stop-words. Following this, data preprocessing methodology was heterogenous between studies with most utilizing a combination of standard text preprocessing techniques (stemming, tokenization). Word embedding was the most common feature extraction method.

### NLP classification models

Twenty studies addressed falls document classification (either detection or prediction) and were subdivided into overlapping groups of binary (13 studies), multiclass (7 studies), and multi-label (2 studies) classification tasks (Table 3). Multi-class/label classification tasks required more complex algorithms. One study addressed this through the use of binary classifiers in ensemble learning, which demonstrated one-vs-one (OvO) ensembles being more effective compared to one-vs-all (OvA) ensembles (micro-averaged F1-score 0.783 vs 0.759). Despite this, CNNs performed better than traditional ML classifiers (F1 score 0.962 vs 0.88) in these complex classification tasks.

**Table 3.** Model architecture and performance of best performing classification models in included publications.

Reference	Classification task	Model architecture	Input features	Feature extraction	Minority class	Class rebalancing method	Internal validation method	Model comparison method	F1 score (95% CI)	AUC (95% CI)	Comparator
Anzaldi et al.	Multi-class Multi-label	Rule-based	Unstructured	Lexicon recall	Not specified	Not specified	Random sampling	NA	Not specified	Not specified	MAC
Bates et al.	Binary	Classical ML (linear L2- regularized SVM)	Unstructured	Lexicon recall with BoW features	Falls	Class-size adjusted misclassification cost	K-fold cross validation	Simple Comparison	0.935	0.970	MAC
Chen et al.	Multi-class	DL (LSTM with BiLSTM, fully connected layer, dropout, and soft max) with attention mechanism	Both	Word Embeddings (Word2Vec, Paragraph2Vec, Med2Vec)	Falls	Performance metric adjustment	Random sampling	McNemar’s Test	Sentence level: 0.735	Not specified	MAC
Dormosh et al.	Binary	Classical ML (logistic regression)	Both	Word Embeddings (Top2Vec)	Non-falls	Not specified	K-fold cross validation	Delong’s Test	Not specified	0.718 (0708–0.727)	MAC
Fu et al.	Binary	Hybrid (BERT + Rule based)	Unstructured	Lexicon recall with Word Embeddings (GloVE), Tokens	Falls	Not specified	Random sampling	Simple comparison	0.98 (0.97–0.99)	Not specified	MAC
Kawazoe et al.	Binary	Hybrid (BERT + BiLSTM)	Unstructured	Tokens	Falls	Loss function weight modification	K-fold cross validation + time-series split	Net reclassification improvement	0.165	0.851	IRC
Kharrazi et al.	Multi-class Multi-label	Rule-based	Unstructured	Lexicon recall	Not specified	Not specified	Random sampling	NA	Not specified	Not specified	MAC
Liu et al.	Multi-class	Classical ML (RF)	Unstructured	Binary text representation	Severity level 3	Random oversampling	Random sampling + stratified K fold cross validation	Repeated grid search + paired 1-tailed t test	Mean macro-0.737	Not specified	IRC
Luther et al.	Binary	Classical ML (SVM)	Unstructured	Term-document matrix	Non-falls	Not specified	Stratified random sampling	Simple comparison	0.866	Not specified	MAC
Martin et al.	Multi-class	Classical ML (elastic net regression)	Both	Word Embeddings (Word2Vec)	Non-falls	Not specified	K-fold cross validation + time-series split	Bootstrapping	Falls Scaled Brier Score 0.27 (0.24–0.31)	0.97 (0.96–0.98)	MAC
McCart et al.	Binary	Classical ML (SVM)	Unstructured	Word (Word2Vec) and Document Embeddings (PV-DBoW)	Falls	Not specified	Stratified random sampling + Stratified k-fold cross validation	Simple comparison	0.853	0.978	MAC
Nakatani et al.	Binary	Proprietary Software (logistic regression)	Unstructured	Word Embeddings (Word2Vec, Phrase2Vec)	Non-falls	Synthetic minority oversample technique (SMOTE)	Repeated random sampling	Student t-test	Not specified	0.834	MAC
Navathe et al.	Binary	Proprietary software	Unstructured	Lexicon recall	Falls	Not specified	Random sampling	NA	0.967	0.865	MAC
Patterson et al.	Binary	Rule-based	Unstructured	Lexicon recall	Falls	Not specified	Iterative random sampling	Simple comparison	0.939	Not specified	MAC
Shiner et al.	Binary	Proprietary software	Unstructured	Lexicon recall, Named Entity Recognition (NER)	Falls	Not specified	Random sampling	Simple comparison	0.67	Not specified	MAC
Tohira et al.	Binary	Classical ML (SVM)	Unstructured	Tf-idf, unigrams	Falls	Not specified	Random sampling + K-fold cross validation	Bootstrapping	0.852 (0.839–0.865)	Not specified	MAC
Topaz et al.	Binary	Classical ML (RF)	Unstructured	Word Embeddings (Word2Vec and Phrase2Vec)	Falls	Not specified	Not specified	Simple comparison	0.85	Not specified	MAC
Toyabe	Binary	Rule-based	Unstructured	Part of Speech (POS) tagging from incident reports	Falls	Not specified	Time-series split	Simple comparison	1	Not specified	MAC
Wang et al.	Multi-class	Classical ML (SVM RBF) One vs one ensemble with DAG decision-making scheme	Unstructured	Binary text representation (BoW)	NA	NA	K-fold random subsampling	Simple comparison	0.88	Not specified	MAC
Wang et al.	Multi-class	DL (CNN)	Unstructured	Word Embeddings (Word2Vec)	NA	NA	K-fold random subsampling	Simple comparison	0.962	Not specified	MAC

Abbreviations.
BERT: Bidirectional Encoder Representations from Transformers. BiLSTM: Bidirectional long-short term memory network. BoW: Bag of Words. DL: Deep Learning. DAG: Directed acyclic graph. EHR: Electronic Health Records. ICD: International Classification of Diseases. LSTM: Long-short term memory network. ML: Machine Learning. SVM: Support vector machines.

Model architecture varied across the best performing models with classical NLP-ML models being the most common (nine studies). Of these, SVM variants and RF classifiers were the most common. Two studies examined DL models in the form of RNNs and CNNs. Three studies utilized proprietary software (PS) of which the model architecture was unable to be clearly elucidated. Two hybrid models (transformer + rules-based and transformer + RNN) were found in the existing literature and one study demonstrated the improved performance of transformers in conjunction with rule-based (F1-score 0.971 vs 0.963) approaches. Transformers that were examined included BERT (Bidirectional Encoder Representations from Transformers), ClinicalBERT, BioClinicalBERT, and RoBERTa (Robustly Optimized BERT-Pretraining Approach).

Most models utilized unstructured features with three studies combining both structured and unstructured features. It was seen that a combination of both types of input variables did not always lead to improved classification performance. Three studies were validated further on external cohorts with all but one study providing results for internal validation. Data splitting for internal validation was most often performed through random sampling and k-fold cross validation, with variations such as time-series splits and stratified sampling methods also being utilized.

### Classification performance

**Figure 2:**
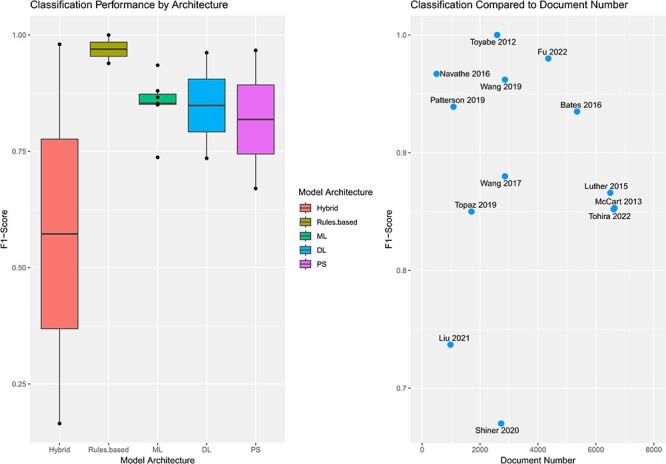
Comparison of classification performance by model architechture and document sample size during training.

Performance metrics (F1-score) were compared across classification studies in terms of identifying falls and related clinical outcomes (Fig. 2). As reflected previously by quality assessment templates, few studies reported confidence intervals for F1-score and area under the curve (AUC) values.

Performance varied across classification models with an overall mean F1-score of 0.826 (0.165–1). Kawazoe’s 2022 model was removed from analysis due to significant differences in F1-score (0.165), leaving 14 models with reported F1-scores for comparison. AUC values were reported in only seven studies. Rule-based models demonstrated the best mean F1-score of 0.970 and was followed by classical ML (0.853), DL (0.8485), and PS (0.8185) models. Document size did not appear to correlate with model F1-score (Fig. 2).

Simple comparison was the most frequently utilized method to compare performance between models; few studies utilized statistical comparison. Statistical methods included: McNemar’s and Delong’s tests, alongside paired *t*-tests.

### Falls risk factor identification

Eight studies examined falls risk factor identification through NLP methods either as an independent model or as part of the input analysis of derived classification models. Bjarnadottir et al. [15] and Topaz et al. [33, 34] were able to identify clinical terms associated with falls, although these terms were not reported in detail for the latter. In the former, a lexicon of established falls risk factors was used to search for term frequencies in nursing notes. These yielded extrinsic and intrinsic falls risk factors which were later classified into environmental, care-process, and technology-related factors. Fong et al. [18], as a precursor to these studies, utilized NLP to match similar clinical documents similar to a known falls report.

Later studies achieved similar results with greater complexity and in the absence of lexicons. This was seen in the study by Nakatani et al. [27] through the generation of 378 morphemes deemed to be significant contributors to falls risk, aligning closely with results outlined by Bjarnadottir. A similar example is seen by Takase et al. [31] who examined the perceptions of nursing staff and their role within fall events. Through the analysis of nursing reports and the development of a co-occurrence term network, they discovered three clusters of commonly recurring topics pertaining to patient, nursing, and environmental falls risk factors.

More recent studies not only enable the automated detection of potential falls risk factors but an evaluation of the extent of contributed risk. Dormosh et al. [17] utilized topic extraction and regression analysis to assign odds ratios (OR) to falls risk factors extracted from clinical documents. Seven topics were found with an OR of greater than 1 and conveyed more significant falls risk, with residential care being the most significant (OR 55.69). Leurs et al. [22] achieved a similar outcome through the examination of single words or combinations with high relative frequency differences between fallers and non-fallers. Their model identified word combinations that were statistically protective against falls (OR <1) and also provided lexical analysis.

## Discussion

### Statement of principal findings

Current studies employing NLP for falls prediction and detection have been applied to four main tasks: falls risk factor identification, known falls detection, future prediction of known falls, and falls severity stratification. The automated identification of falls risk factors has been demonstrated in the studies with the generated terms corresponding with known falls risk factors in the literature. Study characteristics spanned a number of inpatient and outpatient clinical settings, highlighting the ubiquitous nature of falls. It was seen that numerous studies focused on older participants (aged ≥65) and this may be a reflection of the increased prevalence and impact of falls in this population [39].

The choice of clinical documents to review continues to be an area of flexibility with the included studies utilizing a range of different and diverse data types while achieving similar performance. Various forms of clinical documentation may be suitable provided they are sufficiently information rich. In cases where unstructured text data may be lacking, structured clinical data may be a good supplement and can be incorporated into models utilizing mixed structured and unstructured data inputs. The size of the document training sample was not seen as a good predictor of model performance, and the variability in these numbers may point towards the time-intensive bottleneck of data-labelling as part of the NLP-ML pipeline. Data preprocessing and feature extraction methods were heterogenous between studies and sometimes model-dependent, with no one method consistently outperforming the others [40]. Few studies evaluated different feature extraction methods as a way to improve performance [14, 23, 38].

The binary classification of known falls was able to be conducted with reasonable success using rules-based algorithms, traditional ML, DL, hybrid models, and PS. More complex models are present in studies addressing multi-class/label classification problems and are in line with the literature around broader NLP applications towards adverse drug event detection [41], severity stratification, or undifferentiated clinical presentations (i.e. frailty syndromes) of which falls risk is a component [42]. The prediction of future falls was possible and studies indicated that longitudinal data may be of use in this application. Studies also were able to classify the severity of falls from clinical documents with reasonable accuracy. Nil prospective implementation studies were found.

### Strengths and limitations

This review has provided an overview of the literature surrounding NLP applications towards falls in both outpatient and inpatient healthcare settings. This review has a number of limitations. Firstly, the search for articles included articles only in English and relating to healthcare settings. This is a limitation as there is a body of research concerning NLP and falls detection in non-healthcare settings which may help inform future research [43–46]. Secondly, this article attempted basic quantitative analysis of the included publications. These analyses are limited in their interpretation due to the limited number of publications in each group. Thirdly, the quality assessment template used to assess included publications was not fully suitable for ML models utilizing textual inputs, requiring modification. The reporting of ML studies continues to be variable with the development of standardized reporting guidelines (TRIPOD-AI & PROBAST-AI) being underway [47].

### Interpretation within the context of the wider literature

NLP has been employed with a variety of methods to successfully identify and predict falls in healthcare settings. Algorithms utilized have included rule-based, classical ML, DL, and hybrid models to identify fall risk factors and identify falls with reasonable performance. This evidence highlights the potential value that can be gained through the analysis of free-text medical documentation. However, while these studies have proved successful in a variety of settings, at this stage, evidence for successful implementation is lacking. As a consequence of this, outcomes analysis of falls-related consequences via NLP methods remains limited. The investigation of NLP falls applications has also been largely limited to the general and ageing population with specific high-risk populations (i.e. those with gait disturbances) not being yet examined.

### Implications for policy, practice, and research

The identification of risk factors remains highly important in the prevention of in-hospital falls. Individual risk assessment alongside multifactorial interventions have been shown to reduce the rates of falls [48]. As a history of falls may inform future falls risk and prevention strategies [49], the accurate identification of past falls in recorded clinical documents is of increasing importance.

Analysis of free-text medical documentation conveys significant predictive power for fall-related analyses. Insights to guide future efforts to develop algorithms to assist with fall detection and prediction can be obtained through a comprehensive understanding of the existing research. Future research in this area should focus on evaluating the implementation of such models in healthcare settings, as there is a lack of studies assessing how these models will influence patient and system outcomes. For a model to have utility, models will need to be cost-effective and be additive with respect to predictive power relative to the current standard of care [50].

## Conclusions

Through a better understanding of the methods and models utilized to derive NLP models for falls in a healthcare setting, it may be possible to further refine existing algorithms to achieve higher performance in falls risk factor extraction, detection of falls, and automated severity stratification of known falls. The lack of implementation studies indicates a further need to explore the applicability of NLP techniques.

## Supplementary Material

mzad077_Supp

## Data Availability

No new data were generated or analysed in support of this research.
