# Supplementary material for: The use of natural language processing in detecting and predicting falls within the healthcare setting: a systematic review
Source: Int J Qual Health Care. 2023 Sep 27;35(4):mzad077. doi: 10.1093/intqhc/mzad077 (PMC10585351; doi:10.1093/intqhc/mzad077)
Supplement: mzad077_Supp [file mzad077_supp.zip › NLP_Falls_Supplementary.docx]

**Supplementary Information 1: SEARCH STRATEGY**

Key search terms included: “Natural language processing” and “falls” with other related and relevant terms being identified through the MeSH or relevant key terms database.

**MEDLINE (Ovid)**

Search conducted on 12/04/2023

Ovid MEDLINE(R) ALL <1946 to April 11, 2023>

1 "Natural language processing".mp. or Natural Language Processing/ 10206

2 nlp.mp. 3807

3 "text mining".mp. or *Data Mining/ 8690

4 "text classification".mp. 611

5 "information extraction".mp. 1819

6 fall.mp. or Accidental Falls/ 150065

7 falls.mp. 68181

8 falling.mp. 34133

9 faller.mp. 410

10 fallen.mp. 12304

11 "adverse event".mp. 37410

12 "Adverse events".mp. 197986

13 1 or 2 or 3 or 4 or 5 19961

14 6 or 7 or 8 or 9 or 10 or 11 or 12 437195

15 13 and 14 490

Records retrieved: 490

**Embase (Ovid)**

Search conducted on 12/04/2024

Embase <1974 to 2023 April 11>

1 "Natural language processing".mp. or *natural language processing/ 11944

2 nlp.mp. 4861

3 *data mining/ or "text mining".mp. 9338

4 "text classification".mp. 616

5 "information extraction".mp. 1921

6 fall.mp. or falling/ 204854

7 falls.mp. 81104

8 falling.mp. 89848

9 faller.mp. 579

10 fallen.mp. 13468

11 "adverse event".mp. or *adverse event/ 140979

12 "adverse events".mp. 357918

13 1 or 2 or 3 or 4 or 5 23236

14 6 or 7 or 8 or 9 or 10 or 11 or 12 738002

15 13 and 14 735

Records retrieved: 735

**Ovid Emcare <1995 to 2023 Week 14>**

1 "Natural language processing".mp. or natural language processing/ 3969

2 nlp.mp. 1535

3 data mining/ or "text mining".mp. 4870

4 "text classification".mp. 312

5 "information extraction".mp. 914

6 fall.mp. or falling/ 47077

7 falls.mp. 29582

8 falling.mp. 23521

9 faller.mp. 285

10 fallen.mp. 3147

11 "adverse event".mp. or adverse event/ 18421

12 "adverse events".mp. 76916

13 1 or 2 or 3 or 4 or 5 9289

14 6 or 7 or 8 or 9 or 10 or 11 or 12 160675

15 13 and 14 269

Records retrieved: 269

**PubMed**

Search conducted on 12/04/2023

("Natural language processing"[mh] OR "Natural language processing"[tiab] OR "nlp"[tiab] OR "text mining"[tiab] OR "data mining"[mh] OR "text classification"[tiab] OR "information extraction"[tiab]) AND (falls[tiab] OR falls[tiab] OR falling[tiab] OR faller[tiab] OR fallen[tiab] OR "adverse event"[tiab] OR "adverse events"[tiab] OR "accidental falls"[mh])

Records retrieved: 602

**CINAHL**

Search conducted on 12/04/2023

TX ( "natural language processing" or nlp or "text mining" or "text classification" or "information extraction" ) AND ( fall or falls or falling or faller or fallen or "adverse event" or "adverse events" )

Records retrieved: 152

**IEEE Xplore**

Search conducted on 12/04/2023

("All Metadata":"Natural language processing" OR "All Metadata":nlp OR "All Metadata":"text mining" OR "All Metadata":"text classification" OR "All Metadata":"information extraction") AND ("All Metadata":fall OR "All Metadata":falls OR "All Metadata":falling OR "All Metadata":faller OR "All Metadata":fallen OR "All Metadata":"adverse event" OR "All Metadata":"adverse events")Filters applied: Article Type (Journal)

Records retrieved: 24

**Compendex**

Search conducted on 12/04/2023

((("Natural language processing" OR nlp OR "text mining" OR "text classification" OR "information extraction") WN ALL) AND ((fall OR falls OR falling OR faller OR fallen OR "adverse event" OR "adverse events") WN ALL)) - ({ca} WN DT) AND (({chinese} OR {arabic} OR {french} OR {japanese}) WN LA) - {ca} WN DT

Records retrieved: 339

Total records retrieved: 2,611

**Supplementary Information 2**

**DATA EXTRACTION TEMPLATE**

| **Study Characteristics** |
| --- |
| Article Type   - Journal Article - Conference Paper   Year of Publication  Country  Study Type   - Retrospective - Prospective - Derivation - Internal Validation - External Validation - Implementation - Other   Study Setting: Inpatient   - Hospital - Emergency Department - Intensive Care Unit - Other   Inpatient speciality  Please list the medical or surgical speciality/specialities involved if hospital-based study.  Study Setting: Outpatient   - Home - Clinic - Residential Aged Care Facility - Primary Care - Ambulance   Study Setting: Database/Dataset  Select one option.  Yes  No  Database/Dataset Name:  Centres Involved   - Single-Centre - Multi-Centre   Number of Centre's Involved: |
| **Participants** |
| Inclusion Criteria  Exclusion Criteria  Justification  Number of Participants:   - Faller - Non-Faller - Total   Fall Definition: Was the definition of a fall provided?   - Yes - No   Fall Definition: If Yes, please detail here.  Fall Type  Was the study examining a particular type of fall?   - Yes - No   Fall Type:  If yes, please outline here |
| **Data Sources** |
| Data Type  What type of clinical data did this study make use of?   - Structured - Unstructured   Structured Clinical Data  Select all that apply:   - Patient Demographic Information - Laboratory/Blood Test Results - ICD-9 codes - ICD-10 codes - Structured EMR Fields - Structured Incident Report Fields - Other: - Language of structured data (if utilised):   Unstructured Clinical Data  Select all that apply.   - Incident Reports - Medical Progress Notes - Nursing Notes - Discharge Summaries - Imaging Reports - Other   Historical Information  Was any of the information/data sources utilised from previous admissions?   - Yes - No   Language of unstructured data (if utilised):  Justification of data sources:  Predictor Variables  How was the selection of predictor variables determined?  Expert Knowledge (investigators decide that these variables will contribute to the model based on known literature)  Model Derived(algorithms decide that these factors contribute best to the NLP task and thus use them in the final model to derive the output)  Select all that apply:   - Expert Knowledge - Model derived - Other   Data Accrual Period:  Document Sample Size & Fall Capture Rate (TRAINING SET)  The fall capture rate is the percentage of each document type with KNOWN falls. This may be used to identify the best data source (may only be relevant in documents with known falls occurring).   - Document Number (n) - Containing Falls (n) - Fall Capture rate (%) - Incident Reports - Medical Progress Notes - Nursing Notes - Discharge Summaries - Imaging Reports - Other: Specify in comments - Total   Document Sample Size & Fall Capture Rate (TEST SET)   - Document Number (n) - Containing Falls (n) - Fall Capture Rate (%) - Incident Reports - Medical Progress Notes - Nursing Notes - Discharge Summaries - Imaging Reports - Other (please specify in comments) - Total   Annotation Methodology  Number of Annotators   - Single - 2 or more - Not specified   Annotator Knowledge/Role  What was the experience level or roles of the annotators?  Select all that apply.  If uncertain, please specify in the other field.   - Student - Medical Doctor - Nursing Staff - Domain Expert - Other   Inter-annotator agreement  Number = score (often as a number between 0 to 1). Please put NA and do not fill out the rest of the table if this is not outlined by the study.  Method of calculating the agreeance is often Cohen's Kappa. Please put "Not Specified" if the study does not list what method was used.   - Agreement - Number - Method |
| **NLP-ML Model** |
| Falls Task  What was the model intended to do?  Falls risk factors = deriving common words/terms associated with falls (most often text-mining studies)  Falls detection is detecting known falls mentioned in clinical texts.  Falls prediction is detecting future falls based on training with retrospective clinical data from BEFORE when this fall occurred.  Falls severity is grouping known falls into a defined severity rating group.  Falls impact is determining the consequences of known falls. Examples may include morbidity/mortality, economic impact and secondary injuries (i.e. fracture).   - Falls Risk Factors - Detection - Prediction - Severity Stratification - Impact   NLP Task  Select all that are relevant.   - - Binary classification (fall or no-fall)   - Multi-class (more than 2 options)   - Multi-label (a single document can be given multiple labels, i.e. both falls and severity)   - Information Extraction: text mining studies deriving falls risk factors   - Technical NLP   - Other   Note: Clustering studies were excluded as part of this review.  Data preprocessing  Here we will outline any text pre-processing approaches utilised to remove noise from the data.  Some examples include:   - Removing punctuation, stop words, irrelevant digits, white space - Converting text to lower case - Tokenisation - Stemming - Lemmatisation   If possible, list approach and how it was done:  Feature Extraction  Select all that are appropriate   - Lemmatisation - POS Tagging - Ontology/Lexicon retrieval - Term/phrase frequency (tf-idf) - Bag of Words - Word Embeddings - Named Entity Recognition (NER) - Linguistic inquiry and word count (LIWC) - N-grams - Sentiment analysis - Topic models (LSA/LDA) - Other   Word Embedding Method:  If relevant, please specify which word embedding method was used.  Common examples include:  - Word2Vec  - GLOVE  - FastText  - ELMo  - BERT  Model Architecture  Select all broad categories that are relevant and then pick from below regarding specifics.  All studies with models incorporating multiple elements will be known as hybrid models.   - Rule-based - Query/search Based - Classical ML - DL - Proprietary software - Other   ML Models ONLY  Select all that apply, and were investigated in the study.   - Linear regression - Logistic regression - SVM - Random Forest - Naive Bayes - Maximum Entropy - Decision Tree - K-nearest neighbours - Topic Modelling - Other   DL Models ONLY  Select all that apply.  RNN variants may include LSTM, Bi-STEM, Bi-GRU, standard RNN   - RNN - CNN - Feed forward NN - Fully connected NN - Other   RNN Variants ONLY  If RNN, please specify which variants were explored:  Attention Mechanism  If a DL model, does it include an attention mechanism?  Please select NA if not relevant.   - Yes - No - NA   Class balance  Select one which applies.   - Imbalanced - Balanced   Minority class:  Method for Imbalanced Classes  If imbalanced classes, what was the method for dealing with these?  Oversampling methods often involve the duplication of information from the minority class  Under sampling involves removing information of the majority class  Select all that apply or specify in other.   - Random under-sampling - Random over-sampling - Tomek Links - Synthetic minority oversampling technique (SMOTE) - NearMiss - Cost-Sensitive Training (Penalised models) - Weight modification on loss function - Bias initilisation - Adjustment of performance metric - Other |
| **Performance and Evaluation Measures** |
| Datasets  Select all that apply.   - Training: main dataset that is fed into model so that it can learn the data patterns - Validation: dataset that is used to understand the performance of the model in comparison to different models and hyperparameter choices - Testing: Final evaluation of accuracy and performance   Internal validation method  How was the data split into training, validation and testing sets?   - Random: Dataset is shuffled, samples are picked randomly based on a determined percentage ratio - Stratified sampling: Often used for imbalanced classes, distribution of classes in each dataset is preserved. - K-fold cross validation: separatuing dataset into multiple non-overlapping sets - Time series split: Separating the data by time (i.e. all before or after this point in time) - Other   Best performing model  What was the best performing model?  Method of comparison to find best performing models  How did they compare their own models during tuning to find the optimal?  Performance Metrics (classification models ONLY)   - Accuracy - Precision - Recall (Aka Sensitivity) - Specificity - PPV - NPV - F-measure (aka F1 score) - AUROC   Model Comparators  If relevant, what was the performance of the NLP-ML algorithm compared against?   - Manually annotated corpus - Initial reporter classification   Other  Key findings of query based studies  Key findings of text-mining studies |
| **Discussion** |
| - Limitations & Generalisability - Discuss any limitations of the study (such as nonrepresentative sample, few events per predictor, missing data).   Implications   - The potential clinical use of the model and implications for future research.   What was the impact of this study?  Challenges   - Are there planned future studies to be done? - What is preventing this from being more widely implemented? |

**QUALITY ASSESSMENT TEMPLATE**

| **TITLE (1)** |
| --- |
| Identify the report as introducing a predictive model (Y/N) (If text mining study, accept introducing as a text mining study) |
| **ABSTRACT (5)** |
| Background (Y/N) |
| Objectives (Y/N) |
| Data sources (Y/N) |
| Performance metrics of the predictive model or models, in both point estimates and confidence intervals (Y/N) (if text mining study, accept reporting of significant text related to objective) Do not accept if only point estimates or confidence intervals present, both must be there |
| Conclusion including the practical value of the developed predictive model or models (Y/N) |
| **INTRODUCTION (4)** |
| Identify the clinical goal (Y/N) |
| Review the current practice and prediction accuracy of any existing models (Y/N) |
| State the nature of study being predictive modelling, defining the target of prediction (Y/N) (if text mining study, accept stating the study being a text mining study  and defining the context in which terms are found in relation to (e.g. falls risk factors)  instead of the "target of prediction") |
| Identify how the prediction problem may benefit the clinical goal (Y/N) |
| **METHODS (31)** |
| Identify the clinical setting for the target predictive model. (Y/N) |
| Identify the modelling context in terms of facility type, size, volume, and duration of available data. (Y/N) |
| Define a measurement for the prediction goal (per patient or per hospitalization or per type of outcome). (Y/N) Mark as NA if diagnostic study. |
| Determine that the study is retrospective or prospective (Y/N) |
| Identify the problem to be prognostic or diagnostic. (Y/N) (for text mining studies, accept identifying the problem to be a risk factor identification study) |
| Determine the form of the prediction model (Y/N) (1) classification if the target variable is categorical (2) regression if the target variable is continuous (3) survival prediction if the target variable is the time to an event. (4) text mining |
| Explain practical costs of prediction errors (eg. implications of underdiagnosis or overdiagnosis). (Y/N) |
| Defining quality metrics for prediction models. (Y/N) (if text mining study, mark as NA) |
| Define the success criteria for prediction  (eg. based on metrics in internal validation or external validation in the context of the clinical problem). (Y/N) Mark as NA for text mining studies |
| Identify relevant data sources (Y/N) |
| State the inclusion and exclusion criteria for data. (Y/N) |
| Describe the time span of data and the sample or cohort size. (Y/N) |
| Define the observational units on which the response variable and predictor variables are defined. (Y/N) |
| Define the predictor variables.  Extra caution is needed to prevent information leakage from the response variable to predictor variables. (Y/N) |
| Describe the data pre-processing performed, including data cleaning and transformation. (Y/N) |
| Remove outliers with impossible or extreme responses; state any criteria used for outlier removal. (Y/N) (Mark as NA for studies utilising textual data as predictor variables) |
| State how missing values were handled. (Y/N) |
| Describe the basic statistics of the dataset, particularly of the response variable.  These include the ratio of positive to negative classes for a classification problem  and the distribution of the response variable for regression problem. (Y/N) (if text mining study, mark as NA) |
| Define the model validation strategies.  Internal validation is the minimum requirement;  external validation should also be performed whenever possible. (Y/N) (if text mining study, mark as NA) (if impact analyses or extension of previously developed model, then mark as NA) |
| Specify the internal validation strategy.  Common methods include random split, time-based split, and patient-based split. (Y/N) (if text mining study, mark as NA) (if impact analyses or extension of previously developed model, then mark as NA) |
| Define the validation metrics. For regression problems, the normalized root-mean-square error should be used.  For classification problems, the metrics should include sensitivity, specificity, positive predictive value,  negative predictive value, area under the ROC curve, and calibration plot. (Y/N) (if text mining study, mark as NA) (if impact analyses or extension of previously developed model, then mark as NA) |
| For retrospective studies, split the data into a derivation set and a validation set.  For prospective studies, define the starting time for validation data collection. (Y/N) (if text mining study, mark as NA) (if impact analyses or extension of previously developed model, then mark as NA) |
| Identify independent variables that predominantly take a single value (eg, being zero 99% of the time). (Y/N) (Mark as NA for studies utilising textual data as predictor variables) |
| Identify and remove redundant independent variables. (Y/N) (Mark as NA for studies utilising textual data as predictor variables) |
| Identify the independent variables that may suffer from the perfect separation problem. (Y/N) See https://www.jmir.org/2016/12/e323/ for perfect separation problem (Mark as NA for studies utilising textual data as predictor variables) |
| Report the number of independent variables, the number of positive examples,  and the number of negative examples. (Y/N) Accept number of positive and negative falls cases  (if text mining study, mark as NA) |
| Assess whether sufficient data are available for a good fit of the model.  In particular, for classification, there should be a sufficient number of observations in  both positive and negative classes. (Y/N) Mark NA unless classification or prediction study. |
| Determine a set of candidate modelling techniques (eg. logistic regression, random forest, or deep learning).  If only one type of model was used, justify the decision for using that model. (Y/N) (if text mining study, accept description of text mining algorithm(s)) |
| Define the performance metrics to select the best model. (If text mining study mark as NA) (Y/N) If only a single model evaluated, accept defining the performance metrics |
| Specify the model selection strategy.  Common methods include K-fold validation or bootstrap to estimate the lost function on a grid of candidate  parameter values.  For K-fold validation,  proper stratification by the response variable is needed. (If text mining study mark as NA) (Y/N) Mark as NA for studies not utilising internal validation |
| For model selection, include discussion on  (1) balance between model accuracy and model simplicity or interpretability, and  (2) the familiarity with the modelling techniques of the end user. (If text mining study mark as NA) (Y/N) If only a single model evaluated, accept discussion of advantages and disadvantages of model selected |
| **RESULTS (5)** |
| Report the predictive performance of the final model in terms of the validation metrics  specified in the methods section. (Y/N) (if text mining study, accept reporting the terms found by the text mining algorithm) |
| If possible, report the parameter estimates in the model and their confidence intervals.  When the direct calculation of confidence intervals is not possible,  report nonparametric estimates from bootstrap samples. (Y/N) (Mark as NA for text mining study) |
| Comparison with other models in the literature should be based on confidence intervals. (if text mining study, accept comparison between other terminology derived) (Y/N) Mark as NA if no comparisons were made |
| Interpretation of the final model. If possible, report what variables were shown to be predictive of the response variable.  State which subpopulation has the best prediction and which subpopulation is most difficult to predict. (Y/N) (if text mining study, mark as NA) |
| Inclusion of classification error analysis (if not supervised classification model, mark as NA) |
| **DISCUSSION (6)** |
| Report the clinical implications derived from the obtained predictive performance.  For example, report the dollar amount that could be saved with better prediction.  How many patients could benefit from a care model leveraging the model prediction? And to what extent? (Y/N) (if text mining study, accept exploration of its impact) |
| Discussed assumed input and output data format (Y/N) |
| Discussed potential pitfalls in interpreting the model (Y/N) |
| Discussed potential bias of the data used in modelling (Y/N) |
| Discussed generalizability of the data (Y/N) |
| Report unexpected signs of coefficients, indicating collinearity or complex interaction between predictor variables (Y/N) (if textual model, accept discussion of predictor variables and contributions towards model) |
| **TOTAL (52)** |

**Supplementary Information 3: Quality Assessment**

**
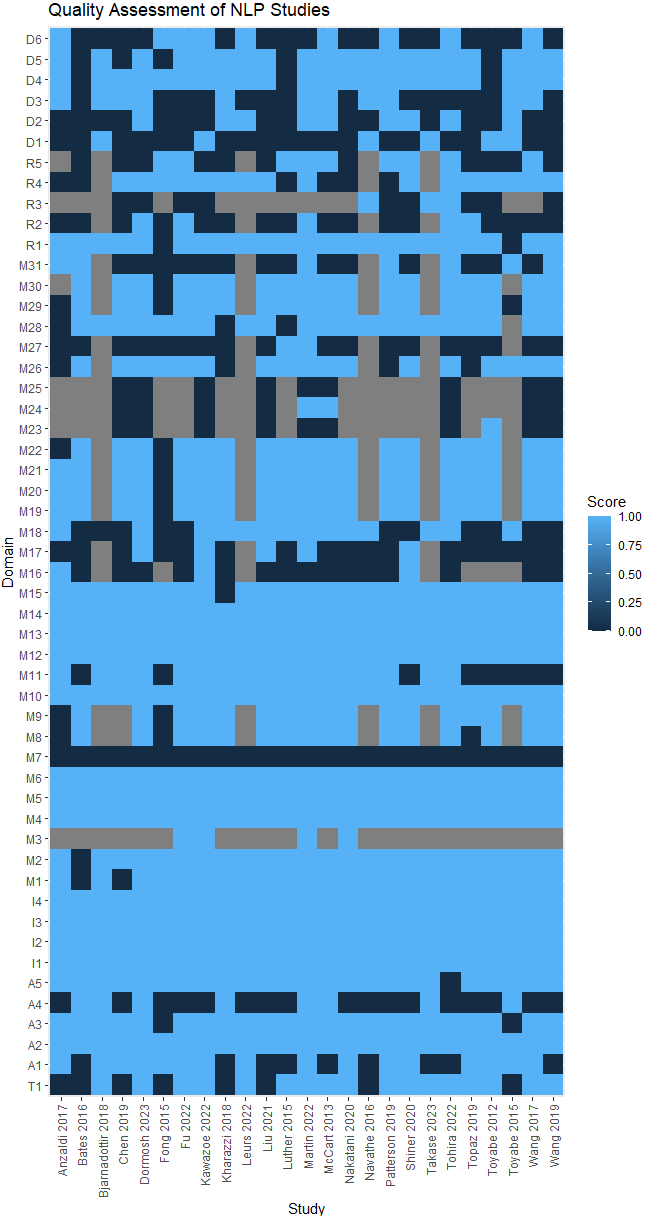
**

**SI3.1:** Graphical and tabular summaries of adherence to quality assessment items

Key: (1) Blue = Y, (2) Navy = N, Grey = NA

**Supplementary Information 3: Quality Assessment (Continued)**

**SI3.2:** Comparison of study quality over time

**Supplementary Information 4: DATA SIZE**

| **Data size** | **No. studies (%)** |
| --- | --- |
| <200 | 0 (0) |
| 200-500 | 2 (7.7) |
| 501-1000 | 1 (3.8) |
| 1001-2000 | 2 (7.7) |
| 2001-5000 | 6 (23.1) |
| 5001-10000 | 4 (15.4) |
| >10000 | 7 (26.9) |
| Unspecified | 4 (15.4) |

**SI4:** Training data size stratified by document number and model architecture with summary measures

|  | **Hybrid** | **Rules based** | **ML** | **DL** | **Text Mining** | **Proprietary software** |
| --- | --- | --- | --- | --- | --- | --- |
| **Mean** | 3030.5 | 20474.6 | 4822.667 | 32210 | 350197 | 291358.75 |
| **STD** | 1875.95429 | 19940.09536 | 2375.653 | 41507.17 | 602631.9907 | 572175.7389 |
